# Array CGH as a first line diagnostic test in place of karyotyping for postnatal referrals - results from four years’ clinical application for over 8,700 patients

**DOI:** 10.1186/1755-8166-6-16

**Published:** 2013-04-05

**Authors:** Joo Wook Ahn, Susan Bint, Anne Bergbaum, Kathy Mann, Richard P Hall, Caroline Mackie Ogilvie

**Affiliations:** 1 Cytogenetics Department, Guy’s and St Thomas’ NHS Foundation Trust, London, SE1 9RT, UK; 2 Cytogenetics Department, GSTS Pathology, London, SE1 9RT, UK

**Keywords:** Array CGH, First line testing, G-banded karyotype analysis, CNV

## Abstract

**Background:**

Array CGH is widely used in cytogenetics centres for postnatal constitutional genome analysis, and is now recommended as a first line test in place of G-banded chromosome analysis. At our centre, first line testing by oligonucleotide array CGH for all constitutional referrals for genome imbalance has been in place since June 2008, using a patient vs patient hybridisation strategy to minimise costs.

**Findings:**

Out of a total of 13,412 patients tested with array CGH, 8,794 (66%) had array CGH as the first line test. Referral indications for this first line group ranged from neonatal congenital anomalies through to adult neurodisabilities; 25% of these patients had CNVs either in known pathogenic regions or in other regions where imbalances have not been reported in the normal population. Of these CNVs, 46% were deletions or nullisomy, 53% were duplications or triplications, and mosaic imbalances made up the remainder; 87% were <5Mb and would likely not be detected by G-banded chromosome analysis. For cases with completed inheritance studies, 20% of imbalances were *de novo.*

**Conclusions:**

Array CGH is a robust and cost-effective alternative to traditional cytogenetic methodology; it provides a higher diagnostic detection rate than G-banded chromosome analysis, and adds to the sum of information and understanding of the role of genomic imbalance in disease. Use of novel hybridisation strategies can reduce costs, allowing more widespread testing.

## Background

Array CGH (aCGH) has a much higher resolution than G-banded chromosome analysis and most cytogenetic departments are now using this approach either as an adjunct to G-banded chromosome analysis, or as a first-line test for selected patient groups [1,2]. The implementation of oligonucleotide aCGH at our centre has been described in a previous paper [3]; this service has been offered since May 2008 using a patient vs patient (phenotype mismatched) hybridisation strategy to minimise costs, an important consideration in a state-funded health service; first line testing by aCGH for all constitutional referrals for genome imbalance has been in place since September 2008. We have now tested a total of 13,412 samples; here, we report on our findings for all samples where aCGH was used as a first line test (n=8,794).

### Patients

Patients were referred from paediatric, neonatal and adult disability populations within our NHS regional area (population ~6M), and from other centres both in the UK and abroad. The median age for the first line testing group was 4 years (range: newborn - 78yrs); referrals were for developmental delay, more specific neurodisability (autism, ADHD, etc.), congenital abnormalities, dysmorphism, or other specific phenotypes (eg café au lait patches).

## Methodology

Genomic DNA extracted from peripheral blood or saliva, or DNA provided by external laboratories, was processed as previously described [3]. Briefly, samples were co-hybridised with other samples mismatched for phenotype and matched for sex (thus halving consumable costs compared with patient vs control). Agilent 4x44k oligonucleotide array platform AMADID 017457 was initially used, replaced in 2010 by an 8x60k platform (AMADID 028469) which included additional probes in regions of clinical interest, and in the pseudoautosomal regions. Analysis was performed using Agilent algorithm ADM-2, threshold 6 and a 3-probe minimum aberration call; a further analysis using ADM-1 was carried out to maximise detection of mosaicism [4]. Imbalances of regions represented in the Database of Genomic Variants [5] in at least three non-BAC based studies were classified benign, and recorded but not reported. All samples with other imbalances were re-tested using G-banded karyotyping, QF-PCR, FISH, custom MLPA [6] or a repeat array.

### Turn-around times and success rates

The average reporting time for first-line tests over the entire period was 21 days from receipt of sample. Hybridisations that did not meet QC metric thresholds (DLRS, % non-uniform outliers, signal intensity and signal to noise) were repeated. Occasionally, it was not possible to complete testing, usually due to DNA degradation. The overall success rate was 99%.

### Imbalances detected

Tables 1 and 2 show the imbalances detected in 8,794 patients tested by first line aCGH. All individuals with features suggestive of Down, Edwards, Patau or Turner syndrome were first tested with QF-PCR [7] and if positive did not proceed to aCGH; hence the prevalence of these syndromes may appear lower in this patient group than in other reports. 25% of patients had CNVs either in known pathogenic regions or in other regions where imbalances have not been reported in the normal population. Of these CNVs, 46% were deletions or nullisomy, 53% were duplications or triplications, and mosaic imbalances made up the remainder; 87% were <5Mb and would likely not be detected by traditional karyotyping. For cases with completed inheritance studies, 20% of imbalances were *de novo.*

**Table 1:** Summary of findings from first line array CGH testing, June 2008 - Sept 2012

	**n**	**%**
**TOTAL FIRST LINE PATIENTS**	**8,794**	
Abnormal	2,218	25% (of total patients)
Normal	6,576	
Completed inheritance studies	1,111	50% (of abnormals)
De novo	226	20% (of completed inheritance)
Inherited	885	80% (of completed inheritance)
**TOTAL IMBALANCES **^**#**^	**2,596**	
Deletions / nullisomy (all chromosomes)	1,182	46% (of all imbalances)
Deletions (autosomes)	1,102	42% (of all imbalances)
Nullisomy (autosomes)	8	<1% (of all imbalances)
Deletions / nullisomy (sex chromosomes)	72	3% (of all imbalances)
Duplications (all chromosomes)	1,240	48% (of all imbalances)
Duplications (autosomes)	951	37% (of all imbalances)
Duplications (sex chromosomes)	289	11% (of all imbalances)
Triplications (all chromosomes)	132	5% (of all imbalances)
Triplications (autosomes)	120	5% (of all imbalances)
Triplications (sex chromosomes)	12	<1% (of all imbalances)
Amplifications	1*	<1% (of all imbalances)
Mosaics (all chromosomes)	41	2% (of all imbalances)
x0~1	5	<1% (of all imbalances)
x1~2	19	1% (of all imbalances)
x1~3	2	<1% (of all imbalances)
x2~3	13	1% (of all imbalances)
x2~4	2	<1% (of all imbalances)
Whole chromosome	79	3% (of all imbalances)
Whole chromosome mosaic	19	1% (of all imbalances)
Reduced copy number >=5Mb	74	3% (of all imbalances)
Reduced copy number <5Mb	1,108	43% (of all imbalances)
Increased copy number >=5Mb	86	3% (of all imbalances)
Increased copy number <5Mb	1,153	44% (of all imbalances)
**PATHOGENIC IMBALANCES **^**#**^	**868**	33% (of all imbalances)
Whole chromosome	79	9% (of pathogenic imbalances)
Syndromic imbalances	225	26% (of pathogenic imbalances)
Susceptibility loci	205	24% (of pathogenic imbalances)
Other pathogenic (private mutations)	359	41% (of pathogenic imbalances)

**#** Patients may carry more than one pathogenic imbalance.

* This patient carried 5 copies in total of a region of chromosome 7.

**Table 2:** Established genomic disorders detected

**OMIM**	**Syndrome**	**n**	**del (x1)**	**dup (x3)**	**trp (x4)**
607872	1p36	2	2	-	-
612474/612475	1q21.1 ^$^	42	20	22	-
600430	2q37	4	4	-	-
609425/611936	3q29	8	2	6	-
194190	Wolf-Hirschhorn	2	2	-	-
123450	Cri du Chat	3	3	-	-
175100	Familial Adenomatous Polyposis 1	1	1	-	-
117550	Sotos	2	2	-	-
194050/609757	Williams-Beuren	14	6	7	1
183600	Split-Hand/Foot Malformation 1	2	2	-	-
610253	Kleefstra	4	4	-	-
194072	WAGR	1	1	-	-
176270/105830	Prader-Willi/Angelman	19	10	7	2
612001	15q13.3 ^$^	26	26	-	-
613406/613406	15q24	2	2	-	-
*	15q26	1	1	-	-
141750	ATR-16	2	2	-	-
**	16p13.11 ^$^	45	13	32	-
136570	16p12.1 ^$^	24	24	-	-
613444	Distal 16p11.2 ^$^	8	8	-	-
611913	Proximal 16p11.2 ^$^	60	35	24	1
247200	Miller-Dieker	9	4 ^	5 ^	-
118220/162500	Charcot-Marie-Tooth/Neuropathy, Hereditary, With Liability To Pressure Palsies	7	4	3	-
182290/610883	Smith-Magenis/17p11.2	10	8	2	-
613675	17q11.2	4	4	-	-
137920	Renal Cysts And Diabetes	10	10	-	-
610443/613533	17q21.31	3	2	1	-
115470	Cat-Eye	1	-	1 ^	-
188400/192430/608363	22q11.2	83	64	19	-
611867	Distal 22q11.2	9	2	7	-
606232	Phelan-Mcdermid	8	8	-	-
308100	X-linked Ichthyosis	11	11 ^^	-	-
312080	Pelizaeus-Merzbacher	1	-	-	1
312750/300260	Rett/MECP2	2	-	2 ^^^	-

**$** Susceptibility locus / incomplete penetrance.

* Tatton-Brown (2009). 15q overgrowth syndrome: a newly recognized phenotype associated with overgrowth, learning difficulties, characteristic facial appearance, renal anomalies and increased dosage of distal chromosome 15q. Am J Med Genet 149:147.

** Hanner (2009). Recurrent reciprocal deletions and duplications of 16p13.11: the deletion is a risk factor for MR/MCA while the duplication may be a rare benign variant. J Med Genet 46:223.

**^** This patient was mosaic.

**^^** all cases were x0 copy nullisomy in males.

**^^^** all cases were x2 copies duplication in males.

The most common findings in our dataset were imbalance in the 22q11.2 deletion syndrome region (OMIM 188400, 192430, 608363; 83 cases), and deletion or duplication of the 16p11.2 autism susceptibility locus (OMIM 611913, 614671; 60 cases). Size of imbalances ranged from <25kb to whole chromosomes, with most pathogenic, syndromic and susceptibility locus imbalances being submicroscopic, whereas “private” imbalances ranged from <25kb to 105Mb, (69% <5Mb). All these diagnostic findings can be viewed on the UCSC Genome Browser via http://bbgre.org.

### Interpretation

The size of imbalances with potential clinical significance generally correlated with severity of phenotype, although there were exceptions. For instance, a ~7Mb duplication (4p15.2p15.1(23,365,794-30,530,905)x3) was found in two siblings, only one of whom had a clinical phenotype. It therefore seems unlikely that the duplication alone was causative in the affected sibling. Towards the other end of the size scale, a deletion of 157kb that included part of SALL1 and no other genes was found in an infant with microcephaly, ear tags and imperforate anus; SALL1 mutation is associated with Townes-Brocks syndrome (OMIM 107480), consistent with the referral indication. These examples demonstrate the potential pitfalls of using arbitrary size cut-offs and the need for careful consideration of gene content of unbalanced regions when interpreting aCGH data.

### Inheritance studies

We were only able to complete inheritance studies for 50% of patients with imbalances; 20% had imbalances that had arisen *de novo*. Inheritance patterns may be considered key to clinical interpretation of aCGH findings; however, it has become clear that simple rules cannot be used [8]. For instance, of our de novo findings, 8% (21/226) did not appear to be associated with the patient’s clinical features; some included no genes or regulatory elements and were therefore unlikely to be clinically significant. Detection of benign de novo CNVs is unsurprising considering estimates for the de novo CNV mutation rate in the normal population [9]. Conversely, at least 20% of inherited imbalances represented susceptibility loci, and were therefore considered to be clinically significant; the clinical status of the carrier parent was generally not known. Penetrance of phenotype associated with these susceptibility loci is often variable; for instance, the 15q13.3 deletion syndrome imbalance has been reported with different clinical presentations within the same family [10].

### Structural information

aCGH does not give information on the location in the genome of, for instance, duplicated regions, or on the structure of chromosomes. However, patterns of imbalance can be used to deduce this information in some cases; for instance, terminal deletion of one chromosome with duplication of terminal material of another chromosome is indicative of a derivative chromosome, and G-banded chromosome analysis and/or FISH for the parents is recommended in these cases. “Inv dup dels” can also be deduced (e.g. 5p15.33p14.1(148,243-27,385,955)x1,5p14.1p13.3(27,463,381-31,329,932)x3), as can ring chromosomes (e.g. 18p11.32p11.21(170,229-14,918,854)x1~2,18q22.1q23(61,430,694-76,083,117)x1~2), supernumerary ring chromosomes (8p12q11.1(35,817,703-47,655,281)x2~3) and inversion recombinants (e.g. 5p15.33p15.2(148,243-13,743,977)x1,5q35.2q35.3(172,591,725-180,617,107)x3). When aCGH is used as the first line test, cultured material from patients may not be available for immediate confirmation of any suspected structural rearrangements by karyotyping or FISH. The correct interpretation of array findings is therefore critical, as this will inform which follow-up studies are most appropriate.

For apparently *de novo,* non-LCR-mediated, interstitial imbalance, karyotype or FISH analysis of the parents is also necessary to exclude a balanced insertional translocation, the presence of which would carry an approximately 50% risk for the parents in future pregnancies. Nowakowska et al. [11] report a frequency of approximately 2.1% of insertional translocation among families of patients with apparently de novo CNVs. In our experience, parental samples for chromosome rearrangement studies are rarely provided; we were able to complete FISH studies on only 12/226 (5%) families with apparently *de novo* findings, and found one parental insertional translocation.

### Incidental findings

These are unavoidable for any whole genome test and can be difficult to deal with clinically, especially with late onset conditions and cancer-susceptibility genes where little may be known of the prevalence or penetrance of clinical features associated with imbalances. In collaboration with our clinical oncology colleagues, we identified 58 genes, imbalance for which was considered likely, based on published studies, to confer increased risk of malignancy [12]. 80 patients in the cohort reported here had imbalance for one of these genes; these imbalances were reported with a recommendation for family studies and genetic counselling. In addition, we have chosen to highlight any other incidental findings where there is a possibility that they may have a significant impact on the health of a patient.

### Balanced rearrangements

Despite the increase in resolution and the higher diagnostic yield associated with aCGH testing, there may be concern that without visualisation of chromosomes by traditional cytogenetic techniques, balanced rearrangements will not be detected. These rearrangements may disrupt gene function without causing any loss of coding material, and hence may be important diagnostically. However, the prevalence of *de novo* apparently balanced rearrangements associated with abnormal phenotype detected by G-banded chromosome analysis is very low, and some of these may in fact be unbalanced at the submicroscopic level; aCGH testing may reveal this imbalance without the need for karyotype analysis first. The increase in diagnostic yield by the use of aCGH remains of far greater patient benefit than the extremely small number of cases where a balanced rearrangement may disrupt an important gene. Whether in a state-funded or private service, the additional benefit of traditional banded chromosome analysis in addition to aCGH testing is likely to be extremely marginal.

### Summary

This report describes 8,794 first line aCGH tests in a state-funded diagnostic laboratory. To our knowledge, this is the largest cohort of patients to date to be reported using this approach, and the results demonstrate that aCGH is a robust and cost-effective alternative to G-banded karyotype analysis, and provides a higher diagnostic detection rate. Implementation of a patient vs patient hybridisation strategy reduces costs, allowing more widespread testing, and therefore adding to the sum of information and understanding of the role of genomic imbalance in disease.

Experience of the challenges in interpretation and reporting of aCGH results will inform the implementation into clinical diagnostic service of higher resolution technologies such as whole exome and whole genome sequencing.

## Competing interests

The authors declare that they have no competing interests.

## Authors’ contributions

JWA designed and constructed the clinical database and compiled the data. KM led the development of the custom MLPA follow-up service, RPH and AB helped with reporting pathways and SB supervised patient matching protocols. CMO designed the hybridisation strategies and led the diagnostic service. JWA and CMO wrote the paper. All authors contributed to interpretation of the array findings, participated in the diagnostic service, and reviewed the manuscript. All authors read and approved the final manuscript.
